# An end-to-end framework for real-time automatic sleep stage classification

**DOI:** 10.1093/sleep/zsy041

**Published:** 2018-03-26

**Authors:** Amiya Patanaik, Ju Lynn Ong, Joshua J Gooley, Sonia Ancoli-Israel, Michael W L Chee

**Affiliations:** 1 Centre for Cognitive Neuroscience, Neuroscience and Behavioral Disorders Program, Duke-NUS Medical School, Singapore; 2 University of California, San Diego School of Medicine, San Diego, CA

**Keywords:** scoring, EEG spectral analysis, computational neuroscience, computerized analysis, deep learning

## Abstract

Sleep staging is a fundamental but time consuming process in any sleep laboratory. To greatly speed up sleep staging without compromising accuracy, we developed a novel framework for performing real-time automatic sleep stage classification. The client–server architecture adopted here provides an end-to-end solution for anonymizing and efficiently transporting polysomnography data from the client to the server and for receiving sleep stages in an interoperable fashion. The framework intelligently partitions the sleep staging task between the client and server in a way that multiple low-end clients can work with one server, and can be deployed both locally as well as over the cloud. The framework was tested on four datasets comprising ≈1700 polysomnography records (≈12000 hr of recordings) collected from adolescents, young, and old adults, involving healthy persons as well as those with medical conditions. We used two independent validation datasets: one comprising patients from a sleep disorders clinic and the other incorporating patients with Parkinson’s disease. Using this system, an entire night’s sleep was staged with an accuracy on par with expert human scorers but much faster (≈5 s compared with 30–60 min). To illustrate the utility of such real-time sleep staging, we used it to facilitate the automatic delivery of acoustic stimuli at targeted phase of slow-sleep oscillations to enhance slow-wave sleep.

**Statement of Significance**

Sleep comprises different physiological stages, with each stage having a unique neurophysiological signature. Traditionally, staging is performed visually by trained sleep technologists. The process is costly and time consuming. In the present work, we utilized recent advances in machine learning, to develop a framework that automatically performs real-time sleep staging. We tested the framework on ≈12000 hr of polysomnography records. Sleep classification was on par with expert scorers across data collected from participants who differed in age and health status. Furthermore, our approach opens up new applications that require real-time sleep-stage dependent interventions.

## Introduction

Polysomnography (PSG) is the primary tool used for quantitatively assessing sleep and involves concurrent acquisition of multiple physiological signals comprising the electroencephalogram (EEG), electrooculogram (EOG), electromyogram (EMG), and electrocardiogram (ECG). Standardized rules for sleep staging using PSG were first laid out by Rechtschaffen and Kales [1] (R&K) in 1968. In 2007, the American Academy of Sleep Medicine (AASM) [2] combined the best available evidence with the consensus of experts in sleep medicine and sleep science to modify the R&K rules, resulting in a higher inter-rater reliability (IRR) of sleep staging than with the R&K system [3]. Under the modified rules, the number of sleep stages was simplified to 5: Wake (W), Stage 1 through Stage 3 (N1, N2, and N3), and REM. Stages 3 and 4 from the R&K rules were collapsed to N3 in the revised scoring criteria, and movement time (MT) was no longer considered as a separate stage.

Numerous automatic sleep stage classification schemes have been proposed and demonstrated, but time consuming and resource intensive human expert review remains the main method by which sleep staging is performed in most clinical and sleep research labs worldwide. Automated systems typically incorporate particular carefully engineered features extracted from PSG data into a classification algorithm. Features that have been extracted from PSG data include spectral power [4–7], band power [4–11], wavelet coefficients [4, 6, 12–14], higher order spectra [15], Hurst exponent [5], auto-regressive model parameters [16, 17], fractal dimension [17], entropy [5, 17, 18], Itakura distance [19], root mean square amplitude [4], peak-to-peak amplitude [4, 6], kurtosis [7, 11, 18], zero crossing [7] amongst others. Classification algorithms include support vector machine [7], Gaussian mixture models [15], artificial neural networks [4–6, 13, 14, 18], learning vector quantization [16], rule and case based reasoning [4, 6, 8], neurofuzzy classifier [19], decision trees [4, 6], linear discriminant analysis [4, 6, 17], extreme learning machine [11], and hidden Markov models [9]. Some methods apply sophisticated artifact correction prior to feature extraction [4, 6]. The accuracy associated with existing automated methods varies from between 75% and 95%. In most instances, the classifier was validated using samples containing less than 100 hr of data [7, 8, 12, 15–18], with some work being supported by as little as 10 hr of validation data [9, 13, 14, 19]. The large variety of methods used, lack of convenient software for clinical users, concern about generalization of the methodology beyond the test samples used to demonstrate proof-of-concept, as well as earlier limitations in computational power, have hindered the broad employment of automated sleep staging systems.

With the growing adoption of artificial intelligence techniques in everyday life ranging from voice recognition to prediction of search preferences, there is a compelling case for reconsidering broad adoption of automated sleep staging to speed up clinical sleep staging as well as to open the door to “real time” applications like targeting memory reactivation [20, 21] or the selective accentuation of slow oscillations in slow-wave sleep (SWS) to augment memory [22–25].

A recent publication in this journal featured one big data approach [11] which used a very large dataset to train and validate an automatic sleep classification system. In contrast to the present work, the approach used was based on tuning multiple, expertly selected statistical EEG features. In contrast, we employed *deep learning* [26] algorithms that engage multiple neural network layers to discover patterns and structure in large datasets. Instead of relying on expertly selected features, deep-learning methods extract features in a data-driven fashion to discover structure and patterns to connect these features to high-level abstract concepts, thereby completely obviating the need for feature engineering.

Deep learning has delivered exceptional performance in applications involving image and speech recognition [26]. Our framework employs a combination of deep and shallow neural networks along with standardized data processing and transport protocols. Its advantages compared with previous methods are as follows: (i) ability to operate on raw PSG data, without relying on artifact and noise correction; (ii) low data transport and processing overheads together with a high level of parallelization, allowing for rapid sleep classification even on consumer-grade hardware; (iii) scoring accuracy that is on par with expert human scorers; and (iv) issuance of a confidence score associated with each scored epoch for review purposes. We also demonstrated a novel application of the framework: to automatically deliver precisely timed acoustic stimulation during slow-wave (N3) sleep, for the purpose of memory augmentation [25].

## Materials and Methods

### Datasets

PSG data were obtained from four independent sources in Singapore and San Diego, USA. The data were acquired according to AASM practice standards and scored by experienced research assistants or registered polysomnographic technologists (RPSGT) according to AASM standards. The data comprised of the following: dataset-1 (DS1) with 1046 PSG records (≈7800 hr) from healthy adolescents (age: 15–19 years), DS2 with 284 PSG records (≈1700 hr) from healthy young adults (age: 21–40 years), DS3 with 210 diagnostic PSG records (≈1600 hr) from patients (age: 10–83 years) of a sleep disorders clinic, and DS4 with 77 PSG records (≈600 hr) from adult patients (age: 47–89 years) with Parkinson’s disease (PD). A combination of DS1 and DS2 was used for training and testing of the classification models. DS3 and DS4 were used as independent validation sets. A total of 11,727 hr of PSG data with 1,403,164 epochs were used for training, testing, and validation. Details of the datasets are summarized in Table 1. All participants provided written consent and data collection and usage were approved by the Institutional Review Board (IRB) of the National University of Singapore (DS1), the SingHealth Centralized IRB (DS2 and DS3), and University of California San Diego IRB (DS4).

**Table 1.** Details of training, testing, and validation datasets

Datasets						
Name	PSG Records (hrs)	N	Source	Scored by	Demographics	Type
DS1	1046* (7777)	120	Cognitive Neuroscience Lab, Duke-NUS Medical School, Singapore	Trained research assistants	Healthy adolescents, age: 15–19 y	75% training, 25% testing
DS2	284* (1749)	52	Chronobiology and Sleep Lab, Duke-NUS Medical School, Singapore	The Siesta Group GmbH, Austria	Healthy adults, age: 21–40 y	75% training, 25% testing
DS3	210 (1590)	210	Sleep Disorders Unit, Singapore General Hospital, Singapore	Trained technicians	Patients with suspected organic and functional sleep disorders, age 10–20 y (4.8%), 21–45 y (42.4%), 46–60 y (33.3%), above 60 y (19.5%)	Validation
DS4	77 (611)	77	Laboratory for Sleep and Chronobiology, University of California San Diego, School of Medicine, USA	Trained technicians	Patients with Parkinson’s disease (PD) with 42% patients additionally classified as having REM-sleep behavior disorder (RBD) and 28% patients as probably having RBD. age: 47–89 y	Validation

Dataset 1 (DS1) and DS2 comprise PSG records from healthy adolescents and adults, respectively. DS3 is obtained from patients from patients in a sleep clinic, whereas DS4 is a sample of patients with Parkinson’s disease. Total 11727 hr.

*includes naps.

### Framework overview

**Figure 1.**
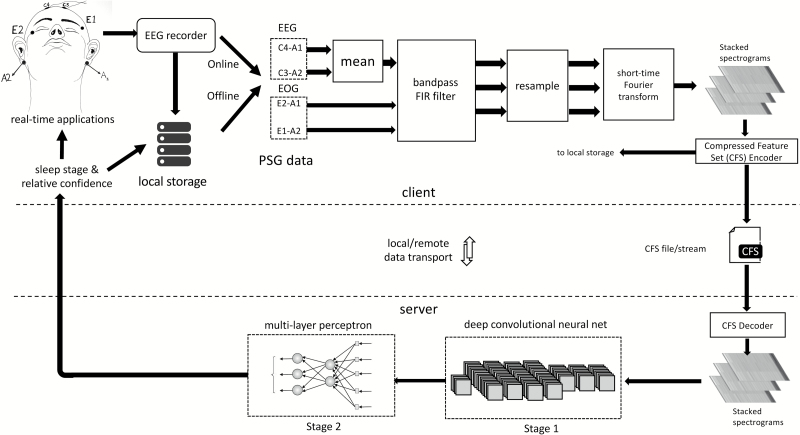
Framework for automatic sleep stage classification. The framework employs a separation of concerns design principle by dividing the task between a client and a server. The client handles data acquisition and preprocessing, while the server handles sleep scoring. The server and client communicate using standardized protocol for high level of interoperability.

The sleep-scoring framework adopted the separation of concerns (SoC) design principle by dividing the task between a client and server module (Figure 1). The client was responsible for interfacing with the EEG recorder (for online sleep classification) or local storage (for offline sleep classification) and preprocessing the data. Offline PSG data were stored in European Data Format (EDF), an open and nonproprietary file format for storage and exchange of digitized PSG data [27]. Two EEG channels (C3-A2 and C4-A1) and two EOG channels (E1-A2 and E2-A1) were used. The four-channel PSG data underwent filtering and resampling operations followed by a short-time Fourier transform, which resulted in a three-channel spectrogram for every epoch of data. The stacked spectrograms were then packed into a compressed feature set (CFS), a standardized binary file format (Figure S2). The CFS format provides an interoperable high-level specification for communication between different implementations of client and server modules. In addition to anonymity, the CFS format afforded significant data compression resulting in far smaller file sizes compared with offline EDF files or raw online data. The software library used to create, stream, and store CFS files from raw PSG data or EDF files is available online (Matlab: https://github.com/amiyapatanaik/cfslib-MATLAB and Python: https://github.com/amiyapatanaik/pycfslib).

The server side module received a CFS file/stream and decoded it back into a stacked spectrogram that was used for sleep classification. The final sleep scores along with their associated confidence scores were sent back to the client in JavaScript Object Notation (JSON), a lightweight open and nonproprietary data-interchange format. A confidence score between 0 and 10 was then assigned, with 0 being low confidence and 10 being very high confidence. This score provides guidance for quality control (QC), i.e. human review of the results of the automated sleep stage classification. A cloud-based implementation of the framework is made available at https://z3score.com. An interface to the sleep classification server module can be found at https://github.com/amiyapatanaik/z3score-api along with sample client code. A modified version of fMRI Artefact rejection and Sleep Scoring Toolbox (FASST [28]) is also accessible from https://github.com/amiyapatanaik/FASST-Z3Score. The toolbox provides an easy to use graphical user interface for the integrated automatic sleep scoring methodology implemented by the sleep classification server.

### Data acquisition and preprocessing: client side

The client side module acquired two EEG channels (C3-A2 and C4-A1) and two EOG channels (E1-A2 and E2-A1) from the EEG recorder or offline storage. Within the preprocessor module, the C4-A1 and C3-A2 channels were averaged, to construct a single EEG channel. If either of the EEG channels was missing, the data were sent directly without averaging. The data were then filtered using a window-based order 50 finite impulse response (FIR) band-pass filter. Computation of filter weights was performed using a Hamming window. The pass-band frequency was 0.3 to 45 Hz for EEG channels and 0.3 to 12 Hz for EOG channels. The filtered data were then downsampled to 100 Hz using a polyphase FIR filter. If the original data were sampled at 100 Hz, the downsampling step was omitted. The data were then divided into 30 s epochs. Each epoch at this stage comprised of 3000 samples per channel. For each epoch, a spectrogram, comprising time-frequency decompositions of the original data, was obtained using a short-time Fourier transform. Specifically, a Hamming window of length 128 with overlap of 29.69 per cent was used and Fourier transform was performed using Fast Fourier Transform (FFT) algorithm. This resulted in a spectrogram with 32 time points (resolution of 938 ms) and 65 frequency points (resolution of 0.7692 Hz). The first 32 frequency bins were considered (corresponding to 0 to 24.6154 Hz) resulting in a 32 × 32 spectrogram. The spectrogram for each channel was then stacked into a three-channel matrix of size 32 × 32 × 3. This resulted in a single stacked-spectrogram per epoch. Spectrograms from all data were stacked together and were encoded to the CFS format (Figure S2). The CFS data stream was transported to the server using Hypertext Transfer Protocol (HTTP).

### Sleep classification: server side

**Figure 2.**
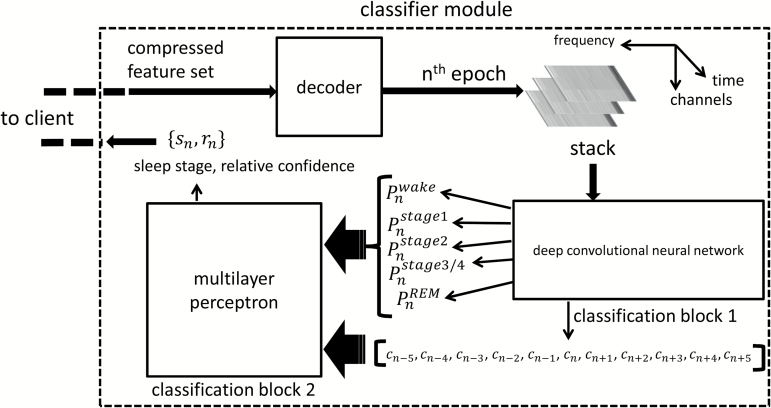
Overview of the classifier module. The classification module receives CFS data from the client and decodes it into stacked spectrograms. The spectrograms are then fed into classification block 1 comprising of a dCNN. The output of the dCNN is fed into a multilayer perceptron in classification block 2. The final sleep stages and associated relative confidence are then sent back to the client.

The server module consisted of a decoder to read the stacked spectrograms from CFS files and two stages of classification blocks (Figure 2). The first classification stage comprised of a 16-layer deep convolutional neural network (dCNN), which took the spectrogram and assigned class probabilities to each sleep stage for that epoch, i.e. for a given spectrogram corresponding to the *n*th epoch, the dCNN, in its final layer generated the probability $\left(p_{n}^{wake},p_{n}^{stage1},p_{n}^{stage2},p_{n}^{stage3/4},p_{n}^{REM}\right)$ that the epoch was classified with a particular sleep stage. The sleep stage with the highest probability was the most probable class (MPC). To numerically compute the MPC, each sleep stage was encoded as a number (1: Wake, 2: S1, 3: S2, 4: S3, 5: REM) and then the MPC was computed as

$$c=argmax\left(p^{i}\right);\;i=1,\ldots ,5.$$

The probabilities for the five possible sleep stages along with the MPC constituted the output of classification block 1.

For offline data, the block-1 classifier was run on all available data. For online data requiring real-time processing, at least five epochs of data were first processed by the block-1 classifier. For any epoch, the output of the block-1 classifier along with five preceding and five succeeding MPC outputs were fed into block-2 classifiers (Figure 2). In online mode, when succeeding MPC outputs were not available, the five preceding MPC outputs were reused. The block-2 classification block consisted of a multilayer perceptron (MLP). The MLP block weighted the succeeding and preceding five MPC outputs along with the current epoch’s block-1 classifications to generate a revised class probability of each sleep stage for that epoch. An MPC block was again used to find the final sleep stage for that epoch. Additionally, a relative confidence score r was computed as

$$r=\min\left(\frac{p^{max}}{p^{secondmax}}-1,10\right),$$

where $p^{max}\;$ is the probability of the MPC and $p^{secondmax}$ is the probability of the second MPC. The score varied between 0 and 10, with 0 signifying very low confidence and 10 signifying very high confidence. By thresholding this confidence score, a portion of the overall data could be marked for review during quality control. The classified sleep stage along with associated relative confidence was sent to the client in JavaScript Object Notation (JSON) over HTTP. The MLP had a total of 445 tunable weights, whereas the dCNN had 177,669 weights. The overall classification blocks have a total of 177,669 + 445 = 178,114 tunable weights. The specifics of architecture for each classification block, along with initialization and training procedures, are discussed in detail in Supplementary Material (Figure S1 and Table S1).

### Novel application: real-time acoustic stimulation

The ability to perform sleep staging rapidly and reliably opens up many novel applications that require stage dependent intervention. For example, boosting SWS, using transcranial stimulation [24] or acoustic stimulation [25] has been shown to enhance declarative memory. Similarly, online detection of REM sleep can be used to time the induction of lucid dreams [29]. In the present work, we demonstrated one such application by automatically delivering precisely timed acoustic stimulation during SWS in real-time.

**Figure 3.**
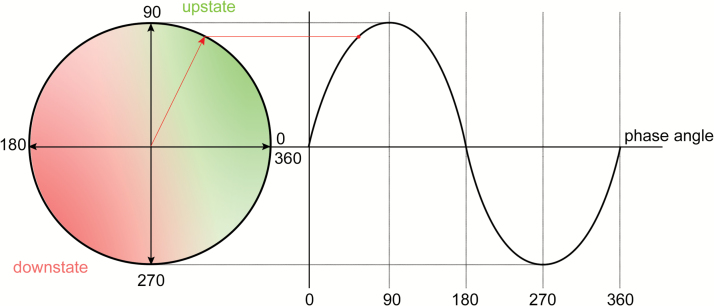
Phase angle of a sinusoidal wave. Acoustic stimulation is most effective when delivered in the up-state (green) and is ineffective when delivered in the down-state (red). Ideally, the stimulus should be targeted for the peak of the wave (90°) while avoiding overshooting.

**Figure 4.**
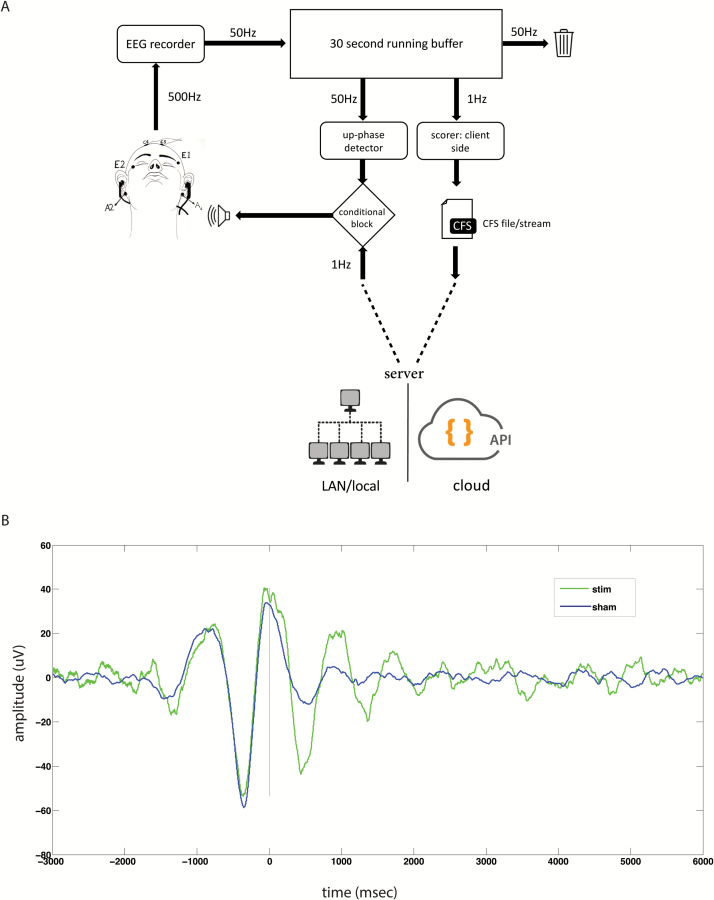
Automatic acoustic stimulation system. (A) Overview of the real-time stimulus presentation system. The scoring and phase tracking were carried out on a running 30 s buffer which was updated at 50 Hz. Scoring was performed using the client–server architecture every second. When conditions based on phase of the EEG wave and sleep stage were satisfied, an auditory stimulus was presented. (B) Average event-related potentials stimulus locked to the first auditory stimulus for a representative participant, who underwent a *stim* and a *sham* session. *Sham* refers to the control condition where phase tracking was carried out but without auditory stimulation. Both sessions were separated by a week.

For effective auditory closed loop simulation of SWS, short bursts of auditory tones must be presented during the up-state of the EEG waveform (Figure 3). Therefore, both sleep stage and phase of the EEG signal must be estimated in real-time. Three EEG channels (F3-A2, C3-A2, and C4-A1) and two EOG channels (E1-A2 and E2-A1) sampled at 500 Hz were acquired in real-time. The F3-A2 EEG channel was used for phase tracing, whereas other channels were used for sleep scoring. The data were fed into a 30 s running buffer (Figure 4A) at 50 Hz. Due to the presence of large DC drifts, a DC-blocking filter [30] with cutoff of 0.03 Hz was applied to the buffer. The running buffer was resampled at 100 Hz using polyphase FIR filters. All filtering was applied in both temporal directions to avoid phase delay caused by filtering. The running buffer was sleep scored once in a second using the framework, whereas phase detection was carried out 50 times a second. Phase tracking of raw EEG signal in real-time is a difficult task. Phase-locked loops (PLL) have been shown to reliably track phase in real-time [31]. However, the implementation and parameter optimization of a PLL-based phase tracker are nontrivial. We used a simple voltage threshold and inflection point–based up-state detection and targeting algorithm (Figure S3). Although the algorithm did not track phase per se, it detected the up-state reliably which was sufficient for our purposes. Auditory tones in the form of 40 ms pink noise were delivered in the form of 2 on and 2 off blocks. During the off blocks, the phase-targeting was carried out but no tones were delivered.

## Results

### Classification accuracy

**Figure 5.**
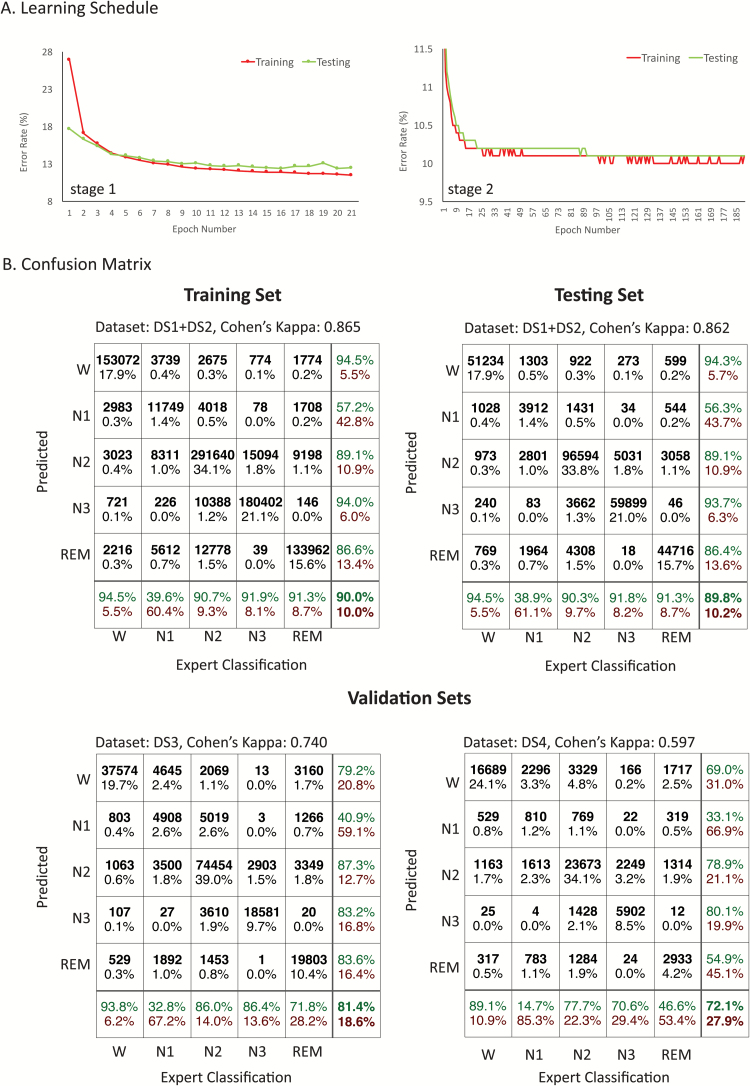
Classification performance of the framework. (A) Training and testing error rates vs. training epoch number for classification blocks 1 and 2. Training was stopped once test error saturated or started increasing. (B) Confusion matrix for training, testing, and validation sets. The training set comprised of 75% dataset 1 (DS1) and DS2, and the testing set comprised of the remaining 25% of DS1 and DS2. DS3 and DS4 constituted independent validation sets.

The classification blocks were trained using 75 per cent of DS1 and DS2, whereas the remaining 25 per cent were used for testing. A single pass through the whole training dataset constituted one training epoch. Training continued until no improvement in accuracy for the testing set was observed (Figure 5A). The block-2 classifier reduced the error rate of the block-1 classifier by 18.4 per cent in the testing set. The overall accuracy was 90 per cent for the training set and 89.8 per cent for the test set, corresponding to Cohen’s kappa (κ) of 0.865 and 0.862, respectively (Figure 5B). This corresponds to perfect agreement according to Landis and Koch’s [32] arbitrary benchmarks for evaluating κ. The highest disagreement between automatic and expert classification was observed for N1 sleep. For other stages, automated and expert classification were in agreement for at least 90 per cent of records.

With validation set DS3, the overall accuracy was 81.4 per cent corresponding to κ=0.740, signifying substantial agreement. Compared with the training and testing set, agreement for REM sleep dropped substantially with automatic classification agreeing with expert classification 71.8 per cent of the time. For validation set DS4, overall accuracy was 72.1 per cent corresponding to κ=0.597, corresponding to moderate agreement. The lower agreement can be attributed to an overall drop of agreement across all sleep stages but particularly for REM, which was expected given that many of the patients with PD also exhibited REM behavior sleep disorder. Classification performance did not improve by increasing depth of the d-CNN any further. Replacing the second stage MLP with a long short-term memory (LSTM) [33] to utilize long-range contextual information resulted in a decrease in classification accuracy in the validation sets.

### Automatic vs expert scoring agreement

**Figure 6.**
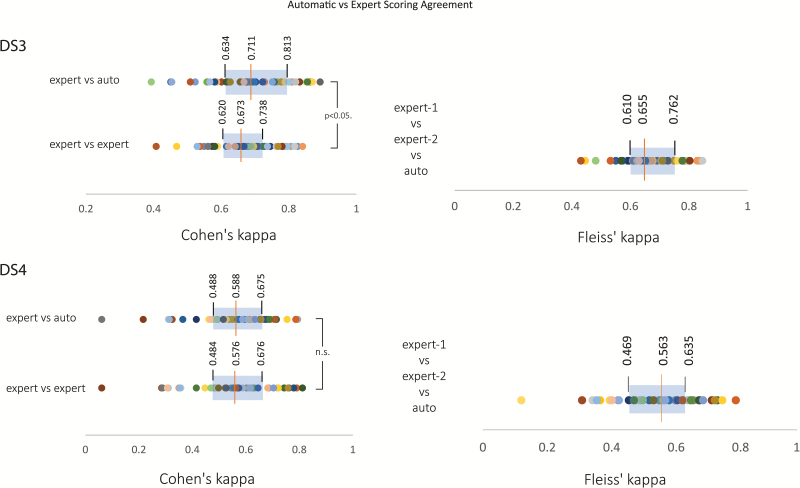
Agreement between automatic and expert scores. Fifty randomly selected polysomnography records from each validation dataset DS3 and DS4 were rescored by an independent organization. This was used to estimate the subject-wise IRR between experts for each dataset as measured by Cohen’s κ and then compared with the computed agreement between automatic scoring and experts. The expert vs automatic scoring agreement was statistically significantly better than expert-expert agreement for DS3 but similar for DS4. The distribution of Cohen’s κ is shown on the left for each validation dataset. The reliability of agreement between the two experts and automatic scoring was assessed using Fleiss’ κ. The box-plot of the distribution of Fleiss’ κ for the 50 records is shown on the right for each validation dataset DS3 and DS4. Within each plot, individual participants are uniquely color coded.

The between experts’ IRR for the validation sets was estimated by rescoring a subset of the data. Specifically, 50 PSG records were randomly chosen from each of the validation sets DS3 and DS4 and were rescored independently by RPSGT-certified sleep technologists from a professional sleep scoring company—SomnoSure, St. Louis, MO—USA. The subject-wise agreement between expert and automatic scoring was slightly lower than the overall agreement rate (i.e. after combining all epochs) for both DS3 (subject wise κ=0.713 vs overall κ=0.740) and DS4 (subject-wise κ=0.560 vs overall κ=0.597). For the rescored subset, a pairwise comparison of subject-wise IRR revealed a higher rate of agreement between automatic and expert scorer compared with the expert–expert scorer (*t*_49_ = 2.320, *p* < 0.05, Figure 6). While for DS4, there was no statistically significant difference in agreement between automatic-expert and expert-expert IRR ($t_{49}=0.013,\;N.S.$). The reliability of agreement between all three raters (two experts and the automatic scorer) was then assessed using Fleiss’ κ for the rescored subset. The median Fleiss’ κ was 0.655 for DS3 and 0.563 for DS4 which correspond to substantial and moderate agreement, respectively (based on Landis and Koch’s [32] arbitrary benchmarks). This was similar to the IRR observed between automatic and expert scorers for the entire dataset.

### Quality control and secondary review system

**Figure 7.**
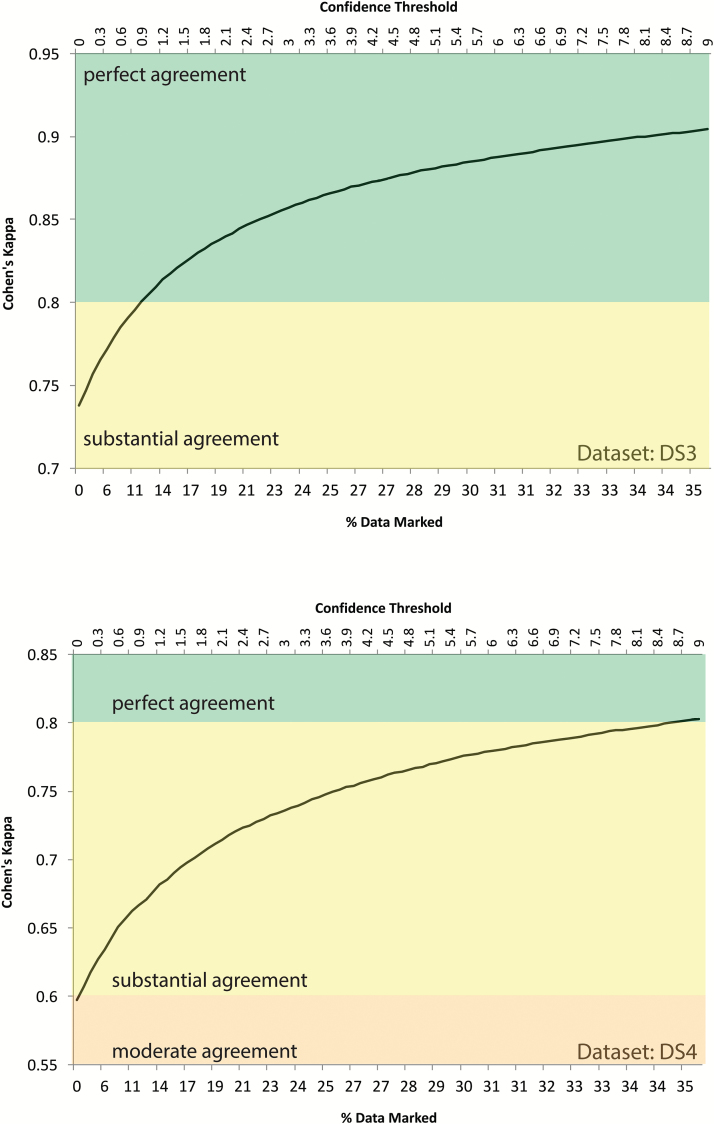
Secondary review system. The automatic sleep scoring framework generated a confidence score in addition to sleep classification for every epoch of data. The confidence score varied between 0 and 10: 0 indicating low confidence whereas 10 indicating high confidence. The secondary review system relied on quickly reviewing epochs with confidence below a set threshold. The figure shows amount of data (bottom *x*-axis) that needed review at a given confidence threshold (top *x*-axis) and the inter-rater reliability as measured by Cohen’s κ on the remaining (nonreviewed) data for both validation datasets DS3 and DS4.

Any machine learning algorithm can yield unexpected results if the input data differ significantly from the training sets. Therefore, notwithstanding the human expert level accuracy of the framework, secondary review is an important aspect of sleep classification. The confidence score associated with each epoch provides guidance for this review whereby only the epochs below a given confidence threshold are recommended for review. For both validation datasets, Figure 7 shows the percentage of data that were marked for review (bottom *x*-axis) for a corresponding confidence threshold value (top *x*-axis) and the auto vs expert IRR value for data that remained unreviewed (*y*-axis). For the validation datasets, a confidence threshold of 1 required 12 per cent of the data to be reviewed for both validation datasets DS3 and DS4 while resulting in perfect agreement for remaining data for DS3 and substantial agreement for DS4 (based on Landis and Koch’s [32] arbitrary benchmarks for evaluating Cohen’s κ).

### Data compression, classification speed, and acoustic stimulation

Using the CFS file format, we achieved an average compression of 16.4× compared with original EDF files (15.97GB compared with 262GB). On a workstation without a GPU (Intel Xeon E5, 3.7 GHz, Quad Core CPU), a single epoch of raw data were scored in less than 5 ms, which works out to ≈5 s for scoring an ≈8.5 hr sleep recording. This included time to convert the raw data to CFS format, transport the data to the locally deployed server, and get back the scored data. This was brought down to sub-1 ms using a mid-range GPU (NVIDIA GeForce GTX-1060).

The framework allowed presentation of acoustic stimulation for boosting SWS fully automatically. Figure 4B shows average event-related potentials stimulus locked to the first auditory stimulus for a representative subject during *stim* and *sham* conditions. *Sham* was a control condition where phase targeting was carried out but no auditory stimulation was given while during *stim,* an auditory stimulus was delivered. A detailed description of the dataset and associated findings of the acoustic stimulation protocol are described in a separate study [34].

## Discussion

Irrespective of the scoring method used (AASM or R&K), the overall IRR among human experts scoring sleep recordings was about 80 per cent (Cohen’s κ = 0.68 to 0.76) [3]. Part of the discrepancy in scoring can be attributed to scorer errors and bias, but most disagreements in scoring were from epochs that cannot be clearly assigned a sleep stage [35]. This results in an upper bound in the performance of any automatic sleep stage classification system. In the present work, accuracy on the training and testing set was higher than IRR values previously reported (~90 per cent agreement between our classification model and expert scores compared with reported expert–expert agreement of ~82% [3]). The higher accuracy can likely be attributed to the classification model mimicking the sleep technicians who scored the training and test datasets instead of attempting to follow a rigid rule-base. However, to be confident of the generalizability of a classifier, its performance should be shown to be on par with expert–expert IRR on a separate dataset, ideally with different characteristics compared with the training samples.

To demonstrate this, we utilized two independent datasets that differed from the training and testing datasets in terms of age, health conditions, geography, EEG recorders used, scoring difficulty, and technicians who scored the data. Validation set DS3 was obtained from patients who visited a sleep clinic. Validation set DS4 included patients with PD and was a considerably more difficult dataset to score. Prior work has shown that IRR is very low for patients with PD [36], with a median κ=0.614. On both datasets, the overall performance of our framework was on par with expert human scorers. This assessment was arrived at through evaluation by an independent set of sleep technicians and reflects achievement of an accuracy level consistent with prior reports [36]. Among the different sleep stages, sensitivity and specificity of N1 detection were lower compared with other stages for both datasets. This is in line with previous observations with human experts [3, 36–38]. These results are promising given that the sleep staging system was trained on young, healthy individuals, and yet, it could accommodate evolving alterations in sleep architecture arising from aging and neurological disease [39].

Our approach is differentiated from prior work, in which training, testing, and validation data were obtained by partitioning the same dataset. Although all machine learning algorithms fit a model to the data, the ultimate goal of any such algorithm is to learn the underlying statistics which are not specific to that particular dataset and to generalize these to data from different sample populations. Even for large datasets, when both training and validation sets are obtained from the same sample, performance on the validation set might not generalize to other datasets as the model might be learning idiosyncrasies specific to that sample. This led us to avoid retraining the algorithm on the validation datasets. Instead, we chose to use confidence scores to provide a way to carry out quality control after automatic sleep classification. Although variations of the framework might further improve performance, the current network architecture represents a good trade-off between maximizing classification accuracy while minimizing network complexity and computation load.

PSG, the gold standard for measuring sleep quantitatively, is a mature technology, and the time-consuming process of sleep scoring has changed little over the years. Burgeoning public interest in the personal measurement of sleep as evidenced by the growing sales of personal sleep/activity tracking devices provides a strong impetus for a robust, easily implemented rapid sleep scoring system. Devices based on actigraphic measures do not reliably quantify duration in each sleep stage [40]. Given the rapidly increasing computing power of personal mobile devices (like smartphones), advances in cloud computing and internet-of-things technology, we anticipate that real-time sleep scoring like what we describe here will open up many novel applications (see for example, Kokoon, https://kokoon.io and Dreem headband, https://dreem.com). Our sleep classification framework is well positioned to take advantage of these developments.

By using a client–server architecture, the computationally heavy classification on the server side is separated from the relatively light preprocessing on the client side. This keeps the technical specifications of the client recording the PSG to a minimum. The CFS file format ties the client and server in an interoperable fashion while significantly reducing data overhead.

In conclusion, our framework provides a practicable, validated, and speedy solution for automatic sleep stage classification that can significantly improve throughput and productivity of sleep labs. It has the potential to play an important role in emerging novel applications of real-time automatic sleep scoring as well as being installed in personal sleep monitors.

## Supplementary Material

Supplementary material is available at *SLEEP* online.

Supplementary Table S1

Supplementary Figure S1

Supplementary Figure S2

Supplementary Figure S3

Supplementary Materials

## Funding

This was not an industry sponsored study. The work was supported by the National Medical Research Council, Singapore (NMRC/STaR/015/2013); the Defence Science and Technology Agency, Singapore (PA/9B12100786), and The Far East Organization.

## Notes

Work Performed: Cognitive Neuroscience Laboratory, Duke-NUS Medical School, Singapore
