# Supplementary material for: An end-to-end framework for real-time automatic sleep stage classification
Source: Sleep. 2018 Mar 26;41(5):zsy041. doi: 10.1093/sleep/zsy041 (PMC5946920; doi:10.1093/sleep/zsy041)
Supplement: Supplementary Table S1 [file zsy041_suppl_supplementary_table_s1.pdf]

layer number	Type	filter support	filter dimension	no. of filters	stride	data size	weights
input	n/a	n/a	n/a	n/a	n/a	32x32x3	n/a
L1	conv	3X3	3	32	1	30X30X32	896
L2	RELU	1X1	n/a	n/a	1	30X30X32	n/a
L3	conv	3X3	32	32	1	28X28X32	9248
L4	RELU	1X1	n/a	n/a	1	28X28X32	n/a
L5	conv	3X3	32	32	2	13X13X32	9248
L6	RELU	1X1	n/a	n/a	1	13X13X32	n/a
L7	conv	3X3	32	64	1	11X11X64	18496
L8	RELU	1X1	n/a	n/a	1	11X11X64	n/a
L9	conv	3X3	64	64	1	9X9X64	36928
L10	RELU	1X1	n/a	n/a	1	9X9X64	n/a
L11	conv	3X3	64	64	2	4X4X64	36928
L12	RELU	1X1	n/a	n/a	1	4X4X64	n/a
L13	conv	4X4	64	64	1	64	65600
L14	RELU	1	n/a	n/a	1	64	n/a
L15	conv	1	64	5	1	5	325
L16	softmax	1	n/a	n/a	1	5	n/a
output	n/a	n/a	n/a	n/a	n/a	6	n/a
