# Supplementary figures and images for: An end-to-end framework for real-time automatic sleep stage classification
Source: Sleep. 2018 Mar 26;41(5):zsy041. doi: 10.1093/sleep/zsy041 (PMC5946920; doi:10.1093/sleep/zsy041)

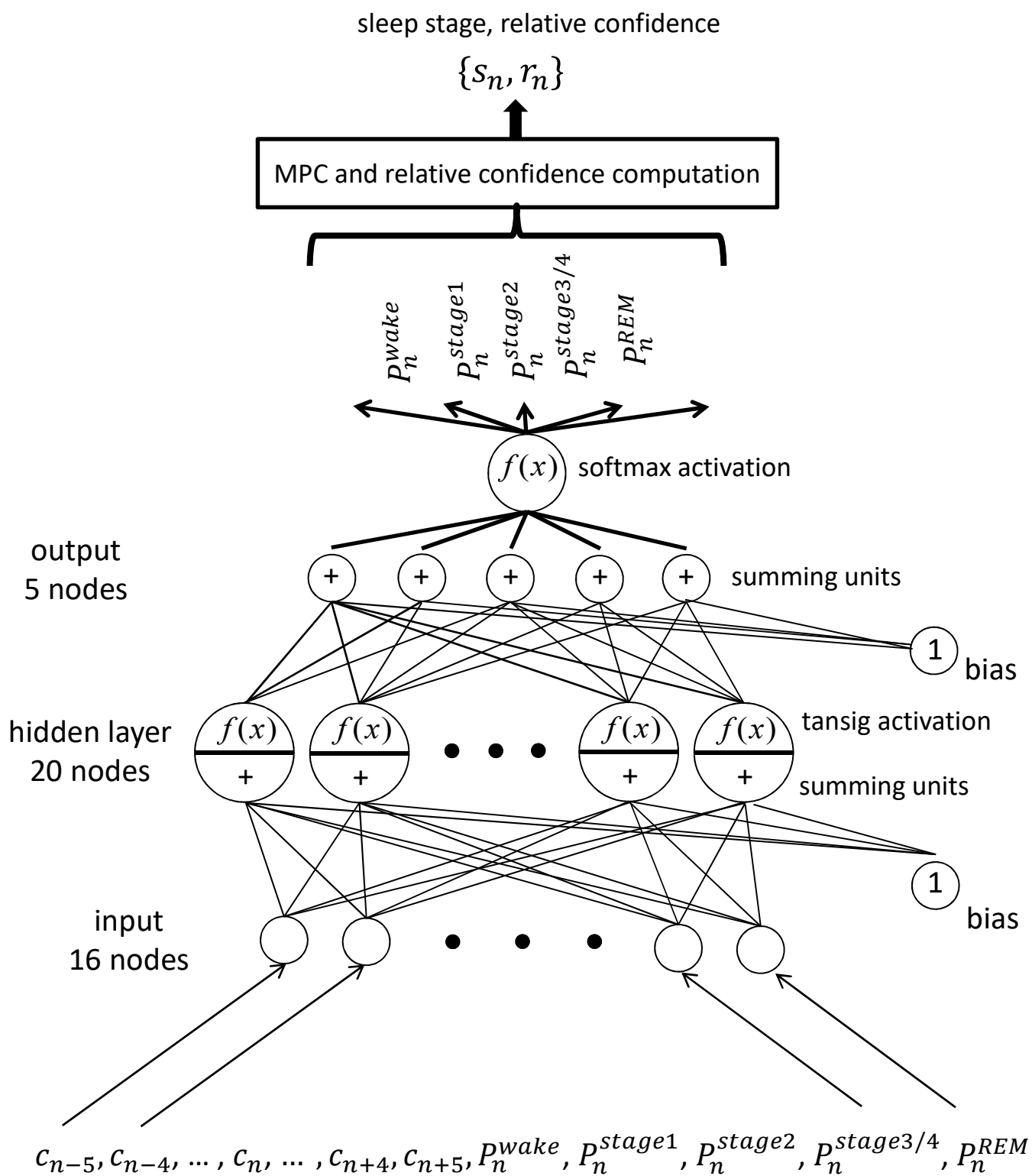

classification block 2

Supplement: Supplementary Figure S1 [file zsy041_suppl_supplementary_figure_s1.pdf]

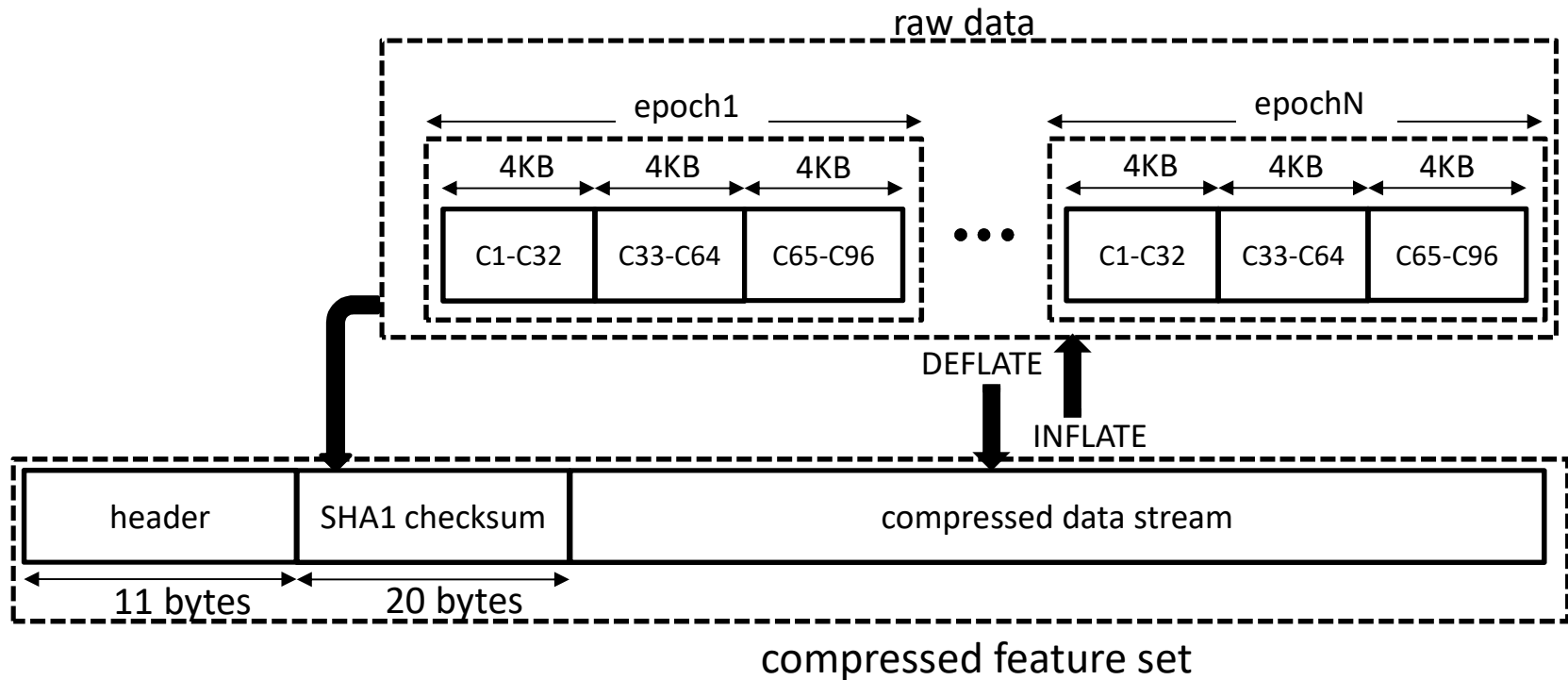

Supplement: Supplementary Figure S2 [file zsy041_suppl_supplementary_figure_s2.pdf]

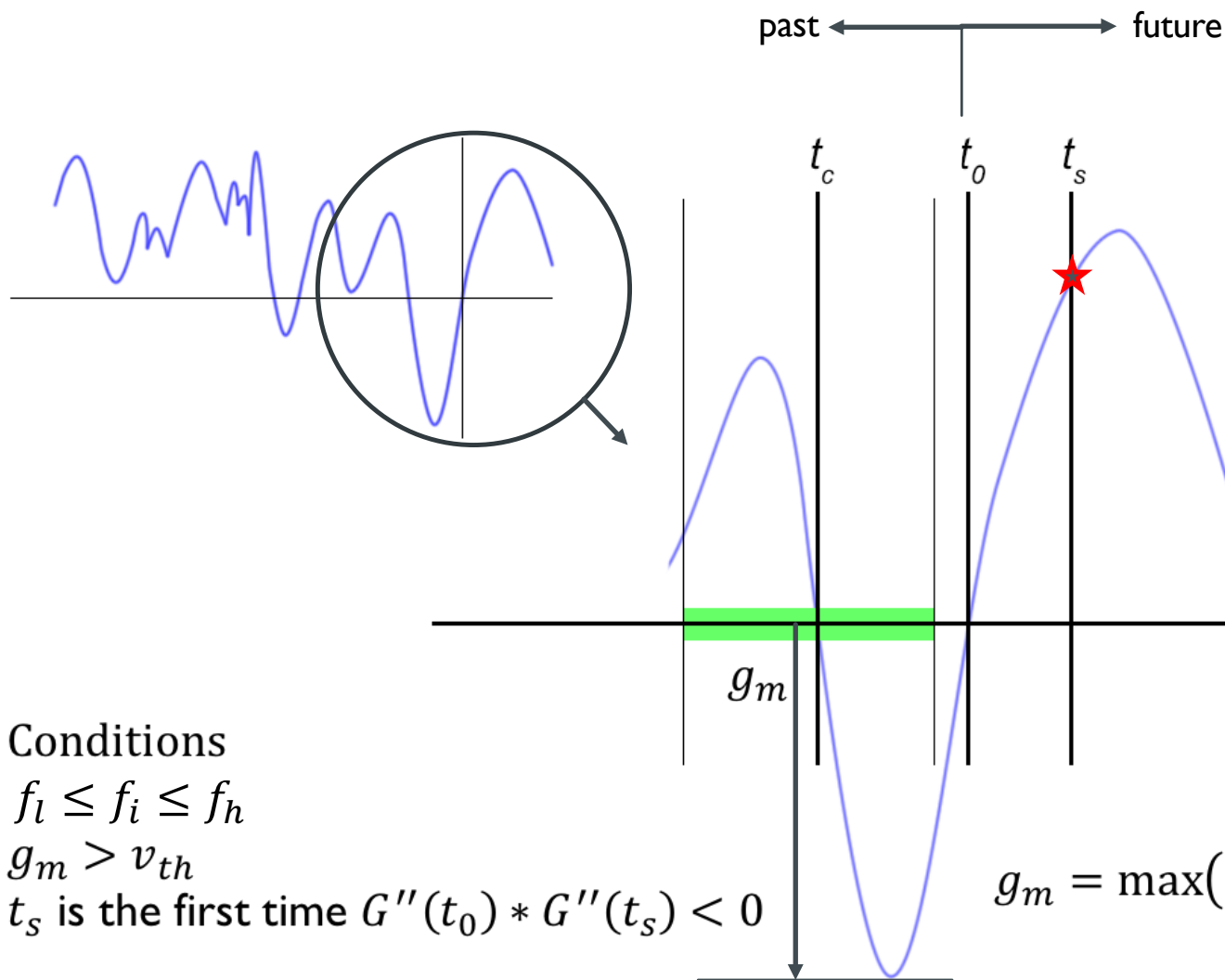

$G(t)$ : EEG waveform

$f_l$ : lower cut-off

$f_h$ : higher cut-off

$t_0$ : current time

$t_c$ : last time  $G(t_0) = G(t_c)$

$$f_i = \frac{1}{2(t_0 - t_c)}$$

Supplement: Supplementary Figure S3 [file zsy041_suppl_supplementary_figure_s3.pdf]
