# Supplementary material for: An end-to-end framework for real-time automatic sleep stage classification
Source: Sleep. 2018 Mar 26;41(5):zsy041. doi: 10.1093/sleep/zsy041 (PMC5946920; doi:10.1093/sleep/zsy041)
Supplement: Supplementary Materials [file zsy041_suppl_supplementary_materials.docx]

**First classification stage: deep convolutional neural network**

The first classification stage comprised of a 16-layer deep convolutional neural network (dCNN), which takes the spectrogram and gave class probabilities of each sleep stage for that epoch. Each layer comprised of multiple convolution filters followed by an activation function layer. The convolution operation was applied on the data from previous layer. The span of each filter is known as its local receptive field. The local receptive field for each filter was moved across the input in steps of $\delta$, also known as stride length. If the input data $I$ is of size $l \times l$ with $n$ channels and each filter $\mathcal{F}$ is of size $d \times d \times n$, then convolution with a stride length of $\delta$ will result in an output image $O$ of size $k \times k$, where $k=\frac{l-d}{\delta}+1$. The value of output at location $i,j$ is given as:

$$O\left( i,j \right)=\sum_{\gamma=1}^{n} \sum_{\beta=1}^{d} \sum_{\alpha=1}^{d} I\left( i\delta+\alpha-1,j\delta+\beta-1,\gamma\right)\mathcal{F(}\alpha,\beta,\gamma)+b; i,j=1,\ldots,k$$

Where $b$ is a bias term. The output of the filter was then sent to an activation function layer. If the activation function is $\theta$, then the output from the activation layer will be

$$O^{'}\left( i,j \right)=\theta(O\left( i,j \right))$$

Each filter has the same weights and bias as it moves across the data. Therefore, each convolution layer with $\omega$ convolution filters of size $d \times d \times n$ will have ${(d}^{2}n+1*\omega$ weights. Due to the nature of the architecture, these convolution computations can be massively parallelized and run on general-purpose computing on graphics processing units (GP-GPU). We use rectified linear unit (RELU) as activation layer for all convolution layers except for the last one. For the last convolution layer, a softmax activation function was used. The RELU function is defined as:

$$f\left( x_{i} \right)=max(0,x_{i})$$

and the softmax function is defined as:

$$f\left( x \right)_{i}= \frac{e^{x_{i}}}{\sum_{n=1}^{N} e^{x_{n}}};n=1,\ldots,N$$

Where, $x_{i}$ is the $i^{th}$ output from previous layer with total of $N$ outputs. The final layer of the network generated class probabilities for each sleep stage. This most probable class (MPC) is then computed as:

$$c=argmax\left( p^{i} \right); i=1,\ldots,5$$

The probabilities for the five possible sleep stages along with the MPC constituted the output of classification block 1. The architecture of the network is shown table S1. We followed an all convolutional neural net architecture. The network has a total of 177,669 trainable weights.

**Second classification stage: multi-layer perceptron**

For any epoch, the output of block 1 classifier along with five preceding and five succeeding MPC outputs were fed in to block 2 classifiers (Figure S1). In online mode, when succeeding MPC outputs were not available, the five preceding MPC outputs were re-used. The Block 2 classification block consisted of a multi-layer perceptron (MLP) with 20 hidden units. The hidden layer is fully connected with the input and output layer. Each node in the hidden layer computed a weighted sum of data, along with a bias and sent it to an activation function. The activation layer for the hidden unit is a hyperbolic tangent sigmoid (tansig) function defined as:

$$f\left( x \right)= \frac{2}{e^{-2x}+1}-1$$

Finally, in the output layer, weighted sum of the results from hidden layer was computed and sent to a softmax activation function, which generates class probabilities. A MPC block was used to find the sleep stage for that epoch.

**Training and Initialization**

The model was trained by randomly initializing all weights according to Glorot uniform initialization^1^. The model weights were then tuned by running the classification blocks on already labeled training data using back-propagation and stochastic gradient descent with Nesterov momentum to minimize the categorical cross-entropy between predicted and actual class labels^2^. Training data for each classification block was shuffled before being fed into the networks in batches. As the model was not very sensitive to the selection of hyper-parameters, the selection of hyper-parameters was based on trial and error. Specifically, we used a batch size of 300 for classification module 1 and 1000 for classification module 2. Learning rate was fixed at 0.001 with momentum of 0.9. Additionally, a learning rate decay of ${10}^{-6}$ was added to classification module 1. We used TensorFlow (Google Inc. Menlo Park, California, USA) to implement our classification modules.

**Compressed Feature Set (CFS) specifications**

To achieve a high level of interoperability, the spectrograms were packed into a standardized compressed feature set (CFS) format. The three-dimensional spectrogram data was vectorized by reading the data column wise (each column corresponds to one-time point) starting from the first channel (C1 to C32 for EEG channel, C33 to C64 for EOG-left channel and C65 to C96 for EOG-right channel). Data from all available epochs were concatenated into a single very long column vector. The spectrogram data was stored in single precision 32-bit floating point number as per IEEE-754 standard. A 20 byte long cryptographic hash of this data was obtained using secure hash algorithm-1 (SHA-1). This hash uniquely identified the spectrogram data without relying on any subject identifiable markers. Additionally, the hash ensured the consistency of spectrogram data as it was sent across the transport channel from client to server. The raw data stream was passed through deflate compression algorithm as described in RFC-1951 specifications (<https://www.ietf.org/rfc/rfc1951.txt>).

This compressed stream constituted the data stream for the CFS format. The first 11 bytes constituted the header of the file, of which the first 3 bytes carried the signature for the file. The signature in HEX is 43, 46 and 53 which reads as ‘CFS’ in ASCII. The next 1 byte carried the file version number. The next 5 bytes carried the dimension of the spectrogram in frequency (1 byte) X time (1 byte) X channel (1 byte) X epochs (2 bytes) format. The last 2 bytes set compression mode and hash set binary flags. When the compression mode was set to 0 the data stream is not compressed. The hash set byte when set to 0 indicates that the SHA-1 hash is not computed and not included in the file. Under normal conditions both these bits would be set. But for online processing, when only one epoch of data was sent to the server at a time, the last 2 bits in the header can be set to 0 and data can be sent without compression and hash. This reduces both computational and data overhead. All binary data was encoded in little-endian format irrespective of underlying hardware architecture. The overview of the format is shown in Figure S2.

**Voltage threshold and inflection point based targeting**

The lower cut-off frequency $f_{l}$ and higher cut-off frequency $f_{h}$ were selected as the valid range of slow wave oscillation. Let the EEG signal to be targeted be $G(t)$ and $t_{0}$ be the current time. If $t_{c}<t_{0}$ was the last time $G\left( t_{0} \right)=G(t_{c})$, then the maximum negative voltage $g_{m}$ is computed as (see Figure S3):

$$g_{m}=\max\left( G\left( t_{0} \right)-G\left( t \right) \right), t_{c}\leq t\leq t_{0}$$

Before computation of $t_{c}$ and $g_{m}$, EEG data was low pass filtered using a second order Butterworth filter with cut-off frequency $f_{h}$ in both temporal directions and the last five samples were ignored to avoid edge distortions caused by filtering. Phase tracking starts if the following conditions are simultaneously satisfied:

- Sleep stage is determined to be N3 with a confidence greater than 5.
- $g_{m}$is greater than threshold voltage $v_{th}$. The threshold voltage could be higher for the first stimulus and lowered for subsequent stimulations.
- The approximate instantaneous frequency $f_{i}$ of the EEG wave was computed as $f_{i}=\frac{1}{{2(t}_{0}-t_{c)}}$ is within valid slow wave oscillation range ($f_{l}, f_{h}$).

Once all three conditions were satisfied, the curvature of the signal in the form of second derivative of the unfiltered EEG signal at $t_{0}$ was computed. For discrete signal $G\left( t \right)$, second derivative at time sample $t_{n}$ is estimated as:

$$G^{''}\left( t_{n} \right)=G\left( t_{n} \right)+G\left( t_{n-4} \right)-G\left( t_{n-1} \right)-G(t_{n-3})$$

While computing the second derivative of the signal, filtering was avoided as filtering causes significant distortion of the edge which adversely affects computation of the curvature. $G''(t_{0})$ is now stored and second derivative is computed every update until time $t_{s}$ when for the first time the second derivative changes sign. In other words:

$$G^{''}\left( t_{0} \right)*G^{''}\left( t_{s} \right)<0$$

A change in sign of the second derivative indicates an inflection point. Acoustic stimulation was delivered at $t_{s}$. Once the stimulus was delivered, phase tracking was stopped for a duration $t_{r}=\frac{1}{{2f}_{i}}$. As a fail-safe if a change in sign was not observed within maximum allowed wait time $t_{max}$, the phase tracking was reset and the stimulus block was marked as invalid.

For the ideal sine wave, the inflection point occurs at 0 degrees. Due to noise and digitization of the signal, stimulation is delivered closer to 45 degrees. This is preferable, as ideally we would like to hit peak of the EEG slow wave at 90 degrees without overshooting. In the present implementation, we fixed the frequency cut-offs $f_{l}, f_{h}$ at 0.5 and 2.0 Hz respectively. The voltage threshold $v_{th}$ was set at 75uV for the first stimulus and 40uV for subsequent stimulations. Maximum wait time $t_{max}$ was set at 3 seconds. The overview of the system is shown in Figure S3.

1. Glorot X, Bengio Y. Understanding the difficulty of training deep feedforward neural networks. In: Aistats; 2010, 2010. p. 249-56.

2. Sutskever I, Martens J, Dahl GE, Hinton GE. On the importance of initialization and momentum in deep learning. ICML (3) 2013;28:1139-47.

**Figure Captions**

**Figure S1: Architecture of classification block 2.** For any epoch, the output of block 1 classifier along with five preceding and five succeeding most probable class outputs were fed in to block 2 classifiers.

**Figure S2: Overview of compressed feature set (CFS) format.** Compressed feature set provides a standardized binary file format for transfer of polysomnographic data to the server for scoring. The CFS format provides significant data compression resulting in far smaller file sizes compared to offline EDF files or raw online data.

**Figure S3: Voltage threshold and inflection point based targeting.** The method utilizes the local shape of the EEG wave to estimate the instantaneous frequency and amplitude of the wave.

**Table S1: Architecture of classification block 1.** The block utilizes a convolutional neural network comprising of 16 layers.
